# Restoring Aesthetics and Function in a Young Boy with Hypomature Amelogenesis Imperfecta: A Case Report

**DOI:** 10.5402/2011/586854

**Published:** 2010-09-21

**Authors:** Engin Ağaçkiran, Emin Caner Tümen, Sema Çelenk, Behiye Bolgül, Fatma Atakul

**Affiliations:** Department of Pedodontics, Faculty of Dentistry, Dicle University, 21280, Diyarbakır, Turkey

## Abstract

Amelogenesis imperfecta has been described as a complex group of inherited conditions that disturbs the developing enamel structure and exists independent of any related systemic disorder. It is a rare dental disease but represents a great restorative challenge for dentists. A 12-year-old boy presented with sensitive, discolored, and mutilated teeth and decreased vertical dimension of occlusion. Direct composite resin restorations were applied to all teeth to modify the occlusion, to restore mild crowding, and to improve aesthetics. The 24-month recall examination revealed no pathology associated with the rehabilitation, and the patient's aesthetic and functional expectations were satisfied. The rehabilitation included multiple anterior and posterior composite resins to eliminate tooth sensitivity, improve the aesthetics and occlusion, and restore function.

## 1. Introduction

Amelogenesis imperfecta (AI) is a hereditary disorder that affects enamel on primary and permanent teeth. It is reported to have an incidence of one person in every 14,000 [1, 2]. Although amelogenesis imperfecta has been categorized into 4 broad groups primarily based on phenotype—hypoplastic, hypocalcified, hypomaturation, and hypomaturation-hypoplastic—at least 15 subtypes of AI exist when phenotype and mode of inheritance are considered [3–8]. Their essential gross features distinguish the hypoplastic and hypocalcified types: in the hypoplastic forms, the enamel does not develop to its normal thickness; in the hypocalcified forms, the enamel thickness on newly erupted teeth closely approaches that of normal teeth, but the enamel is soft, friable, and can easily be removed from the dentin. In contrast to hypoplastic types, the hypomaturation types develop enamel of normal thickness. The hypomaturation forms differ from hypocalcification in that the enamel is harder, with a mottled opaque white to yellow-brown or red-brown color, and tends to chip from the underlying dentin rather than wear away [3, 9, 10]. 

According to the literature, AI patients, regardless of subtype, have similar oral complications: teeth sensitivity, poor dental aesthetics, and decreased occlusal vertical dimension [2]. Other dental anomalies associated with AI include, but are not limited to, multiple impacted teeth, congenitally missing teeth, open occlusal relationship, and taurodontism [8, 11]. 

Historically, patients with AI have been treated with multiple extractions and the fabrication of complete dentures [2, 12–15]. Recently, several studies have illustrated the use of stainless steel crowns, adhesive casting, over denture, porcelain veneers, ceramics, and composite resin veneers to restore dentitions mutilated by severe attrition [12–15]. Besides, the advances in the field of aesthetic dentistry, especially in bonding to dentin, help practitioners to restore function and aesthetics to an acceptable level [9, 16]. 

 This clinical report describes treatment of a 12-year-old boy patient diagnosed with hypomature amelogenesis imperfecta by using direct composite resin laminate veneers.

## 2. Case Report

**Figure 1:**
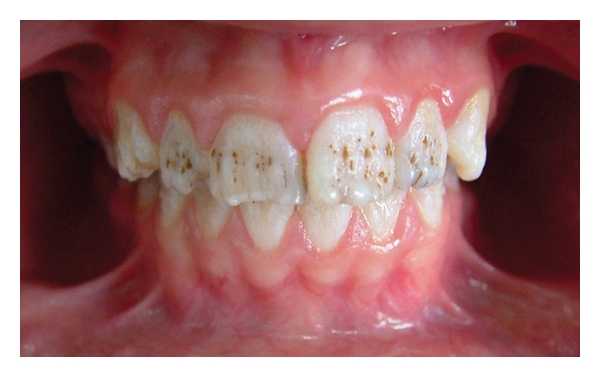
Frontal view (pretreatment).

**Figure 2:**
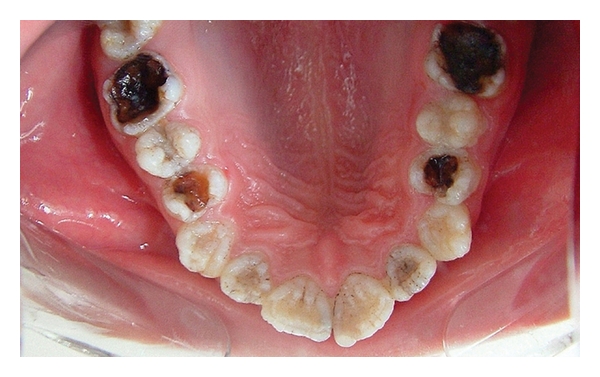
Occlusal view of the maxillary arch (pretreatment mirror view).

**Figure 3:**
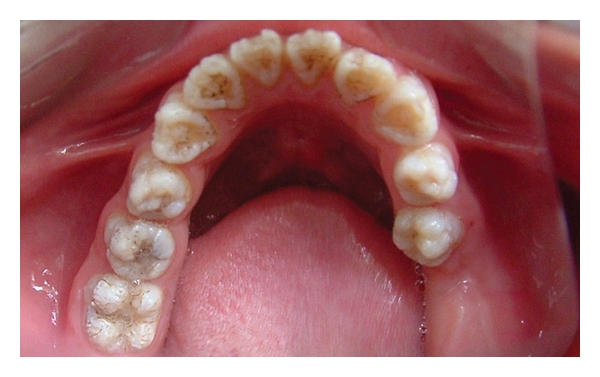
Occlusal view of the mandibular arch (pretreatment mirror view).

A 12-year-old boy was referred to the Department of Pediatric Dentistry, Dicle University, Diyarbakır, Turkey for examination, evaluation and treatment of gross attrition and considerable sensitivity of his teeth. A detailed medical, dental, and social history was obtained. He was both self-conscious and unhappy as regard the appearance of his teeth. Clinical examination revealed that tissue loss affected all teeth. The enamel layer was very thin and Brown (pigmented), the cuspal structure was completely absent in the occlusal portion of the molars which were most severely affected, and enamel pit defects (pigmented stains deep) were present in the anterior teeth (Figures 1, 2, and 3). However, the clinical appearance of cervical and approximal enamel seemed to be normal. It was thought that the patient likely suffered from a hypomature type of AI. The patient's occlusal vertical dimension and rest vertical dimension were assessed. The interocclusal rest space had increased because of attrition of the molars. The exposed dentin was hypersensitive. Clinical and radiographic examination of the patient revealed deep carious lesions in the maxillary four teeth, and the mandibular left first molar had been extracted because of severe attrition and caries approximately 10 months earlier. 

A treatment plan was developed with the following aims: to reduce the reported sensitivity of the teeth, to modify the occlusion, to restore mild crowding and masticatory function, and to improve the aesthetics. The patient was informed of the diagnosis and all treatment plans were discussed with him and his parents. He could not afford prosthetic treatment, including porcelain or ceramic restorations. Therefore, the treatment plan included placement of direct composite resin restorations in maxillary and mandibular anterior and posterior teeth. These materials were chosen because it cost less than ceramic and porcelain restorations and with the hope that it would ensure aesthetic and functional rehabilitation until the patient could cover the cost of porcelain restorations.

**Figure 4:**
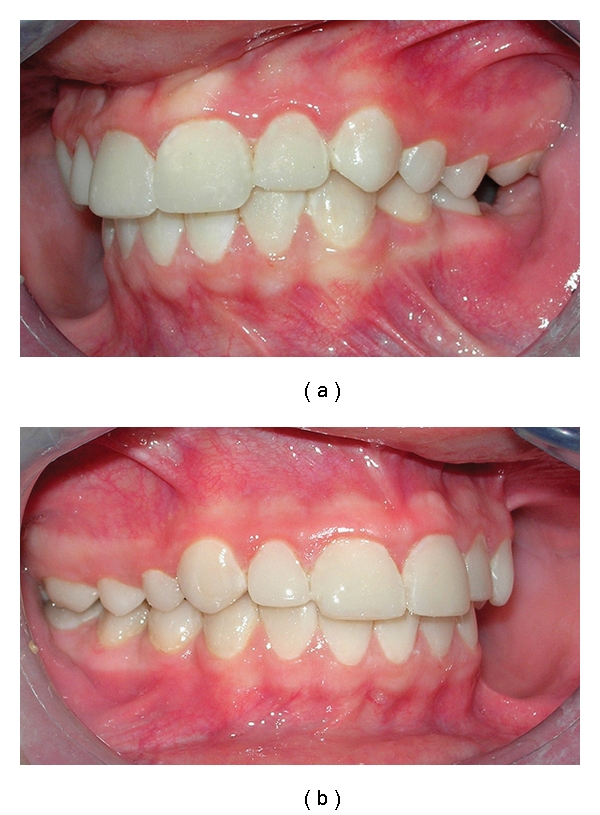
Frontal view of the left and right sides (posttreatment).

**Figure 5:**
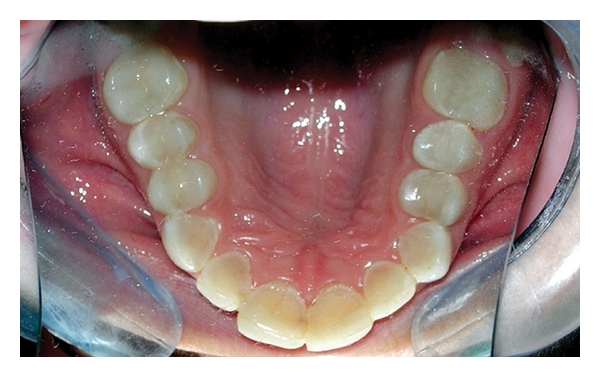
Occlusal view of the maxillary arch (posttreatment mirror view).

**Figure 6:**
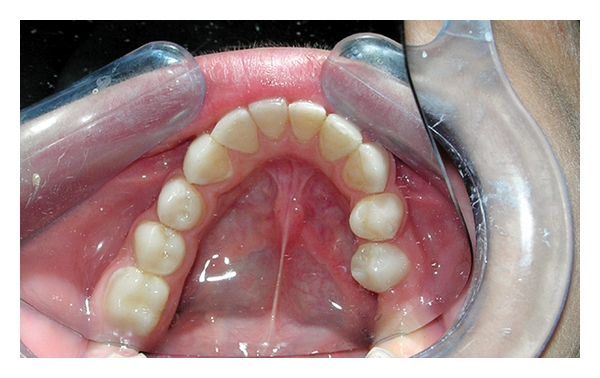
Occlusal view of the mandibular arch (posttreatment mirror view).

Firstly, according to the manufacturer's directions maxillary and mandibular posterior teeth of patient were restored using posterior composite resin (3 M ESPE-Filtek P60, St. Paul, MN, US), after deep caries in maxillary premolar and molar teeth were removed. Later, on the maxillary and mandibular anterior teeth, a 0.5 mm facial reduction was prepared for direct composite resin laminate veneers. In addition, the finish line at the proximal and cervical aspects of the teeth preparations was extended, and a rounded finish line throughout was prepared for the restoration. All teeth preparations were completed without sharp line angles. Maxillary and mandibular anterior teeth of the patient were restored using composite resin (3 M ESPE-Filtek Supreme XT Universal, St. Paul, MN, US) (Figures 4, 5, and 6). 

**Figure 7:**
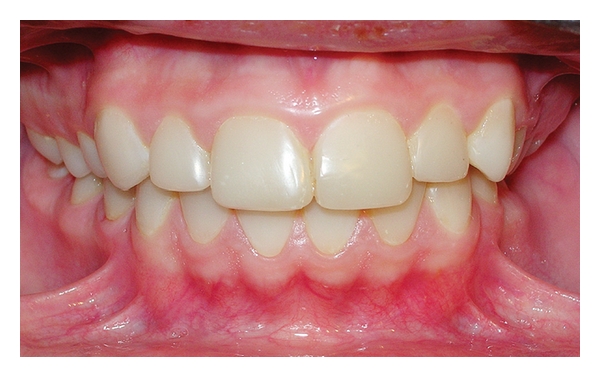
Control view after 24 months.

 The patient was recalled at 2-month intervals. The 24-month recall clinical and radiographic examination revealed no pathology associated with the rehabilitation, and the patient's aesthetic and functional expectations were satisfied (Figure 7).

## 3. Discussion

The term “amelogenesis imperfecta” (AI) describes a diverse group of hereditary conditions primarily affecting the quality and/or quantity of dental enamel. The affected teeth show a soft enamel of normal thickness that chips and wears easily and has a radiodensity similar to that of dentin [9]. The results of clinical and radiographic evaluations indicated that the patient in the present case had hypomaturation form AI. All the teeth are misshapen, and spotted. Occlusion and vertical opening are rapidly affected by attrition. The insufficiency of the enamel makes the teeth extremely sensitive to contact and thermal stimuli. These problems combine to make early diagnosis essential and immediate treatment a necessity, even for the youngest patients [17, 18]. 

In some types of AI, the patient's enamel not only is thin but also may display abnormal mineral content. The axial surfaces may be chalky, weak, and highly susceptible to carious breakdown. Such teeth require complete coverage with preformed crowns until precision cast crowns can be provided in the patient's late teens or early adulthood.

A complex case will challenge any direct composite system. A quality aesthetic direct composite system must contain a wide variety of shades and include multiple opacities to ensure the restorations will mimic natural dentition. As teeth are not monochromatic and the colors come from within, the color must be developed from the inside out. The polish of a quality direct composite system should provide high gloss and should be maintained in the oral environment.

Tulga [19] applied composite resin restorations for anterior teeth to 10-year-old child with AI as provisional treatment. Similarly, in the present case the treatment was completed with direct composite resin. Although this case could have been treated with indirect restorations, direct resin treatment was chosen to preserve tooth structure and for financial reasons. When all the teeth had been restored direct composite resin, the patient' aesthetic complaints disappeared completely, and normal eating habits were established. Temporary restorations provide satisfactory aesthetic and protective features with minimum preparation, made difficult by crown height and lack of enamel in untreated amelogenesis imperfecta, to be performed under excellent conditions. This temporary treatment allows the normal development of the dentition to continue unimpaired and, last but not least, is affordable for the parents.

 It is very important in that it prevents the development of psychological problems arising from the appearance of teeth affected by amelogenesis imperfecta [12]. The shy and taciturn 12-year-old boy first brought forcefully to the department, became a willing and talkative patient. The psychological transformation was spectacular, as was the physiological change, evidenced by a weight gain within weeks.

## 4. Conclusion

This clinical report describes the oral rehabilitation of a 12-year-old boy patient affected by hypomature amelogenesis imperfecta. The treatment plan for cases of AI is related to many factors: the age of the patient, the socioeconomic status of the patient, the type and severity of the disorder, and the intraoral situation at the time the treatment is planned. The rehabilitation included multiple anterior and posterior composite resins to eliminate tooth sensitivity, improve the aesthetics and occlusion, and restore function.
